# MECP2 promotes the growth of gastric cancer cells by suppressing miR-338-mediated antiproliferative effect

**DOI:** 10.18632/oncotarget.9197

**Published:** 2016-05-06

**Authors:** Dongdong Tong, Lingyu Zhao, Kang He, Hongfei Sun, Donghui Cai, Lei Ni, Ruifang Sun, Su'e Chang, Tusheng Song, Chen Huang

**Affiliations:** ^1^ Department of Cell Biology and Genetics, School of Basic Medical Sciences, Xi'an Jiaotong University Health Science Center, Shaanxi, P. R. China; ^2^ Key Laboratory of Environment and Genes Related to Diseases, Xi'an Jiaotong University, Ministry of Education of China, Shaanxi, P. R. China; ^3^ Department of Periodontology, Stomatology Hospital of Xi'an Jiaotong University College of Medicine, Xi'an, Shaanxi, P. R. China; ^4^ Department of pathology, College of Medicine, Xi'an Jiaotong University, Xi'an, Shaanxi, P. R. China; ^5^ Department of Orthopaedics, the Second Affiliated Hospital, School of Medicine, Xi'an Jiaotong University, Xi'an, Shaanxi, P. R. China

**Keywords:** gastric cancer, MECP2, miR-338, BMI1, proliferation

## Abstract

The methyl-CpG-binding protein 2 (MECP2), a transcriptional suppressor, is involved in gene regulation by binding to methylated promoters. We found that MECP2 is overexpressed in gastric cancer (GC), and that *Mecp2* knockdown affects the growth of GC cells both *in vitro* and *in vivo*. MECP2 can directly bind to the methylated-CpG island of miR-338 promoter and suppress the expression of two mature microRNAs, namely, miR-338-3p and miR-338-5p. Furthermore, miR-338-5p can suppress GC cell growth by targeting BMI1 (B lymphoma Mo-MLV insertion region 1 homolog). We additionally found that decreased miR-338-5p expression in GC tissues, relative to normal tissues, was significantly negatively correlated with increased BMI1 expression. Silencing MECP2 can indirectly lead to reduced expression of P-REX2, which has been identified as the miR-338-3p target, as well as BMI1 and increasing expression of P16 or P21 both *in vitro* and *in vivo*. Altogether, our results indicate that MECP2 promote the proliferation of GC cells via miR-338 (miR-338-3p and miR-338-5p)-mediated antitumor and gene regulatory effect.

## INTRODUCTION

Gastric cancer (GC) is the fourth most common malignant cancer and the second leading cause of cancer-related deaths, China is a high incidence area, and alone accounts for 42% of the incidence observed worldwide [1]. Poor prognosis of GC performs 4% to 20% of 5-year-suvival rate because most patients are invariably diagnosed during the advanced stage [2]. At present, studies have focused on the molecular mechanisms observed in GC and uncovered various tumor-related factors such as cytokines, growth factor, and chemokines, which are associated with the proliferation, migration, and invasion of GC cells [3, 4]. However, the regulatory networks involved in GC are complicated and multifactorial, and it is critical to identify molecular targets that hold promise for prognostic and therapeutic strategies.

Genomic methylation is an important topic in cancer research. Cancer initiation and progression is accompanied by global DNA hypomethylation and site-specific CpG island promoter hypermethylation [5, 6]. Methylation of CpG islands present within the promoter region resulted in the silencing of tumor suppressors at the transcriptional level [7], including those encoded by tumor suppressor genes such as *ER, BRCA1*, and *Rb* [8–10]. Interestingly, not only the gene transcripts but also some non-coding RNAs that have emerged as a leading cause of gastric tumorigenesis have been found to undergo cancer-specific inhibition through hypermethylation of promoters [11]. MECP2 is an important constituent of the DNA methylation machinery; it is directly involved in the mediation of epigenetic signals and is quite crucial for neural development. Meanwhile, certain mutations in *Mecp2* can cause Rettsyndrome [12]. MECP2 is thought to be a transcriptional repressor and requires a specific methylated CpG site for preferential binding to DNA [13]. Previous studies, mostly in neurons, have identified many gene transcripts or miRNAs as MECP2 targets [14, 15]. The role of MECP2 in tumor progression regulation has been reported in lung cancer, hepatocellular carcinoma, and osteosarcoma. In addition, MECP2 is involved in cell development, cell cycle, apoptosis, invasion, and migration [16–18]. Although MECP2 is a known link between DNA methylation and transcription of tumor suppressors and might contribute to GC cell growth, there is little knowledge about its role in gastric tumorigenesis.

MicroRNAs (miRNAs) are small, noncoding RNAs, 21~25 nucleotides in length, which are known as master gene mediators because they form the miRNA-induced silencing complex (miRISC) and lead to mRNA instability or degradation [19]. Aberrant miRNA expression is observed in many biological processes such as cell proliferation, cell cycle, apoptosis, invasion, and migration, for example, in case of miR-145, miR-638, miR-27, miR-129, and miR-196b. Depending on the cellular function of certain miRNA targets, miRNAs can behave as oncogenes or tumor suppressor genes. These miRNAs have been identified as tumor suppressors in GC. Interestingly, miR-196b and miR-129 are modulated by methylation in the CpG island [20–24].

Apoptosis-associated tyrosine kinase(AATK) gene is located on chromosome 17 (17q25.3) [25]. Former studies have shown that the role of *Aatk* in anti-tumorigenesis and aberrant *Aatk* expression depends on methylation in the CpG island promoter of *Aatk* [26, 27]. MiR-338(miR-338-3p and miR-338-5p) is generated from an intron of the gene coding for Aatk and both molecules are co-expressed because they share the same promoter. In our previous study, miR-338-3p was shown to act as a tumor suppressor by targeting P-rex2 in GC [28], but the role of miR-338-5p in human GC is still unidentified. In this study, we showed that MECP2 is upregulated in GC and that it increased the proliferation of GC cells both *in* vitro and *in vivo*. MECP2 suppressed miR-338 by directly binding to the methylated CpG island of the promoter, and then indirectly promoted miR-338 targets. Furthermore, we verified the significant downregulation of miR-338-5p expression in GC tissues and explored that it potentially suppressed cell growth by acting as a key suppressor of the proto-oncogene *Bmi1* involved in transcriptional controlling. Our hypothesis is that MECP2 facilitates the growth of GC cells through MECP2/miR-338-3p/P-REX2/AKT and MECP2/miR-338-5p/BMI1/signaling.

## RESULTS

### MECP2 is frequently overexpressed in GC cells and promotes cell growth and proliferation in GC cell lines

**Figure 1:**
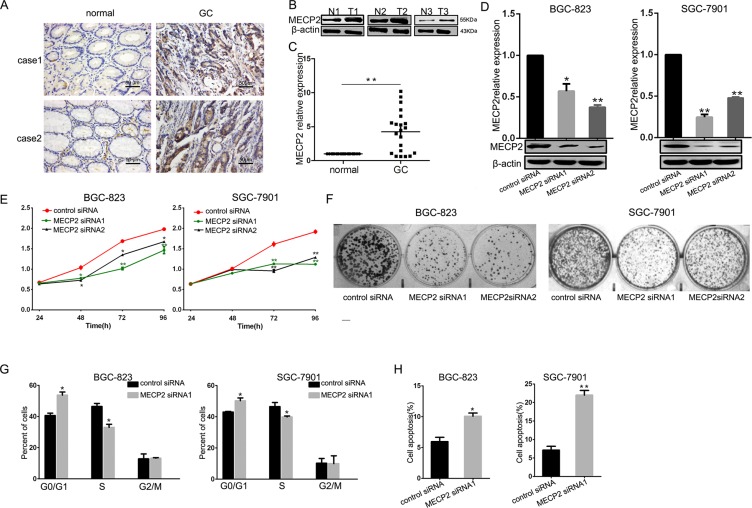
MECP2 is overexpressed in GC samples and effects cell growth and proliferation on GC cell lines *in vitro.* (**A**) MECP2 protein expression level in gastric cancer tissues were measured by IHC staining (Bar = 50 μm), 2 cases were presented. (**B**) Western blot analysis of MECP2 expression in non-tumor gastric (N) and gastric cancer (T) tissues, 3 paired samples were presented. (**C**) qRT-PCR of MECP2 mRNA expression in 21 paired GC and their corresponding nontumorous tissues, The results in (a) are expressed as means ± SD. **P < 0.01. The expression of MECP2 was normalized to β-actin. (**D**) qRT-PCR and western blot analysis of MECP2 in BGC-823 and SGC-7901cells transfected with MECP2 siRNA1, MECP2 siRNA2 or control siRNA, β-actin served as an internal control. Results are expressed as means ± SD *P < 0.05, **P < 0.01. (**E**) At 24, 48 72 and 96 h after transfection with MECP2 siRNA1 and MECP2 siRNA2, BGC-823 and SGC-7901 cell proliferation were determined by the MTT assay. (**F**) The growth of BGC-823 and SGC-7901 cells was detected by colony formation after MECP2 knockdown. (**G**) The histograms for cell cycle distribution of BGC-823 and SGC-7901 cells treated with MECP2 siRNA1 after 48 h based on the flow-cytometric analysis. (**H**) Apoptosis rate of BGC-823 and SGC-7901 cells was examined by Annexin V staining and flow cytometry at 48 h after transfecting with MECP2 siRNA1, representative results of 3 independent experiments were shown. Data shown are mean ± SD value. (*P < 0.05, **P < 0.01, Student's *t*-test).

To demonstratethe potential functions of MECP2 in GC, we determined MECP2 levels by immunohistochemical staining (IHC) and western blot of GC tissues. MECP2 expression was significantly upregulated in GC samples compared to their adjacent normal gastric tissues (Figure 1A and 1B). Further, the results of qRT-PCR for 21 pairs of clinical tissues revealed the same tendency (Figure 1C). MECP2 was markedly overexpressed in GC, which indicates that it may have played the role of an oncogene. To exclude the possibility of off-target effects, we transfected two oligonucleotides of MECP2 siRNA1 and MECP2 siRNA2 in BGC-823 and SGC-7901 cell lines, qRT-PCR and western blot were used to validate the efficiency of siRNA. In addition, MECP2 siRNA1 and siRNA2 sufficiently deregulate MECP2 expression in both cell lines (Figure 1D). Next, MTT (3-(4,5-dimethyl-2-thiazolyl)-2,5-diphenyl-2-H-tetrazolium bromide) assay was used to investigate the effect of MECP2 on the proliferation of GC cells; we found that deregulated MECP2 caused lower proliferation of BGC-823 and SGC-7901 at 48 and 72h after transfection (Figure 1E). The colony formation assay showed that cell growth was inhibited in MECP2 siRNA-transfected BGC-823 and SGC-7901 cells (Figure 1F). This effect can be partially explained by the inhibition of cell growth regulation on MECP2 targeting, such as cell cycle arrest and apoptosis. Therefore, we analyzed BGC-823 and SGC-7901 cells by flow cytometry to study the influence of MECP2 on cell cycle progression; notably, We transfected MECP2 siRNA1 in GC cells and found the arrest of G1/S transition (Figure 1G). Further, annexin V staining verified that MECP2 siRNA1 significantly promotes cell early apoptosis in both GC cell lines (Figure 1H). Parallelly, the knockdown induced by MECP2 siRNA2 showed the same result of MECP2 siRNA1 in cell cycle or apoptosis (Supplementary Figure 1). Based on these investigations, we confirm that MECP2 exerts the effects of an oncogene on G1/S progression and apoptosis, and thus promotes the proliferation of GC cells.

### MECP2 suppresses miR-338 by binding the methylated CpG island of miR-338 promoter

**Figure 2:**
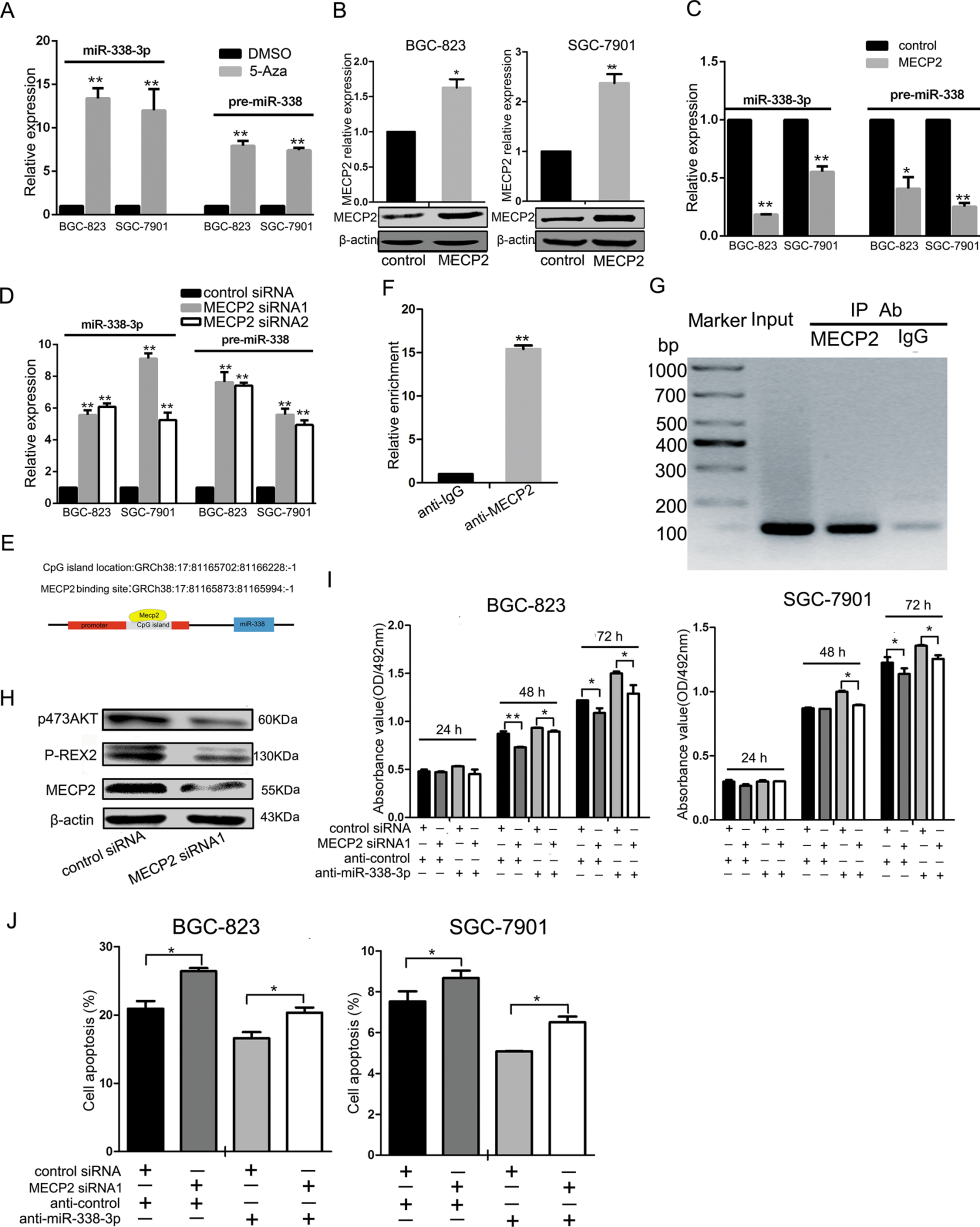
MECP2 inhibited miR-338 expression in gastric cancer cells (**A**) The BGC-823 and SGC-7901 cells were treated with 5-Azacytidine (5-Aza) (5 μmol), the equal volume of DMSO as control, expression of miR-338-3p and pre-miR-338 was measured by qRT-PCR. The expression of miR-338-3p and pre-miR-338 was normalized to U6 RNA. (**B**) qRT-PCR and western blot analysis of MECP2 protein and mRNA levels expression in BGC-823 and SGC-7901 cells were transfected with GV146 vector containing cDNA of MECP2 or control vector. (**C**) The miR-338-3p and pre-miR-338 expression was quantified by qRT-PCR in BGC-823 and SGC-7901 cells transfected with GV146 vector containing cDNA of MECP2 or control vector. (**D**) qRT-PCR analysis of the miR-338-3p and pre-miR-338 expression in BGC-823 and SGC-7901 cells following transfection with two MECP2 siRNAs or control siRNA. (**E**) Sketch of the putative CpG island locus and the MECP2 binding site in human miR-338 promoter. (**F**) ChIP assays were performed with control (rat IgG), anti-MECP2 antibody to determine MECP2 occupancy of miR-338 promoter. (**G**) qRT-PCR analysis was performed with primers spanning predicted CpG island of miR-338. (**H**) Western blot analyses of P-REX2 and phosphorylated AKT(Ser-473) in BGC-823 cells transfected with MECP2 siRNA1 or control siRNA. (**I**) MTT assay was performed to determine the growth of BGC-823 and SGC-7901 cells after cotransfected with MECP2 siRNA1 and inhibitor control or siRNA control and miR-338-3p inhibitor. (**J**) Early Cell apoptosis were detected by Annexin-V/propidium iodide combined labeling flow cytometry in BGC-823 and SGC-7901 cells 48 hours after transfection. Each data represented mean ± SD. *P < 0.05, **P < 0.01, Student's *t*-test.

We treated GC cells with 5-azacytidine (5-Aza) (5 μmol) or DMSO(equal volume) and checked miR-338-3p and pre-miR-338 expression by qRT-PCR analysis after 48 h. miR-338-3p and pre-miR-338 were increased in BGC-823 and SGC-7901 cell lines. (Figure 2A). To further investigate whether MECP2 could suppress miR-338 expression, we constructed MECP2 overexpression vectors, transfected the test and control vectors into GC cells, and examined MECP2 level by qRT-PCR analysis or western blot. Compared to the control vector-transfected cells, mRNA and protein levels of MECP2 were significantly increased (Figure 2B), and miR-338-3p and pre-miR-338 expression was downregulated (Figure 2C). Furthermore, knockdown of *Mecp2* enhanced miR-338-3p and pre-miR-338 expression (Figure 2D). We predicted a CpG island in the promoter located upstream of the miR-338 locus by bioinformatics analysis by the methprimer [29] (Figure 2E). The GC percentage of the predicted CpG island is shown in Supplementary Figure 2. To prove our hypothesis that MECP2 suppresses miR-338 expression, we conducted a ChIP assay using real-time PCR to further validate the involvement of MECP2 in the CpG island of miR-338 promoter. We detected remarkable binding of MECP2 to this region in BGC-823 cells, but no IgG recruitment was observed (Figure 2F and 2G). Given the fact that P-REX2 is a target of miR-338-3p and that it plays the role of an oncogene by inducing Akt phosphorylation [28], we found that MECP2 siRNA1 induced the decreased protein of P-REX2 or P-AKT (Figure 2H) as part of the investigation to determine whether MECP2 could indirectly affect miR-338 target gene. These results suggested that MECP2 inhibited miR-338 expression by occupying a specific CpG island in the miR-338 promoter, and that MECP2 indirectly activated miR-338 target genes.

To determine the role of miR-338-mediated transcription by MECP2, we used anti–miR-338-3p to inhibit miR-338-3p expression, which was cotransfected with MECP2 siRNA1 into BGC-823 or SGC-7901 cells. We found that miR-338-3p silencing rescued the negative effect of MECP2 siRNA1 in GC cells during cell proliferation and facilitated cell early apoptosis (Figure 2I and 2J).

### MECP2 inhibits miR-338-5p; BMI1 is a direct target of miR-338-5p

**Figure 3:**
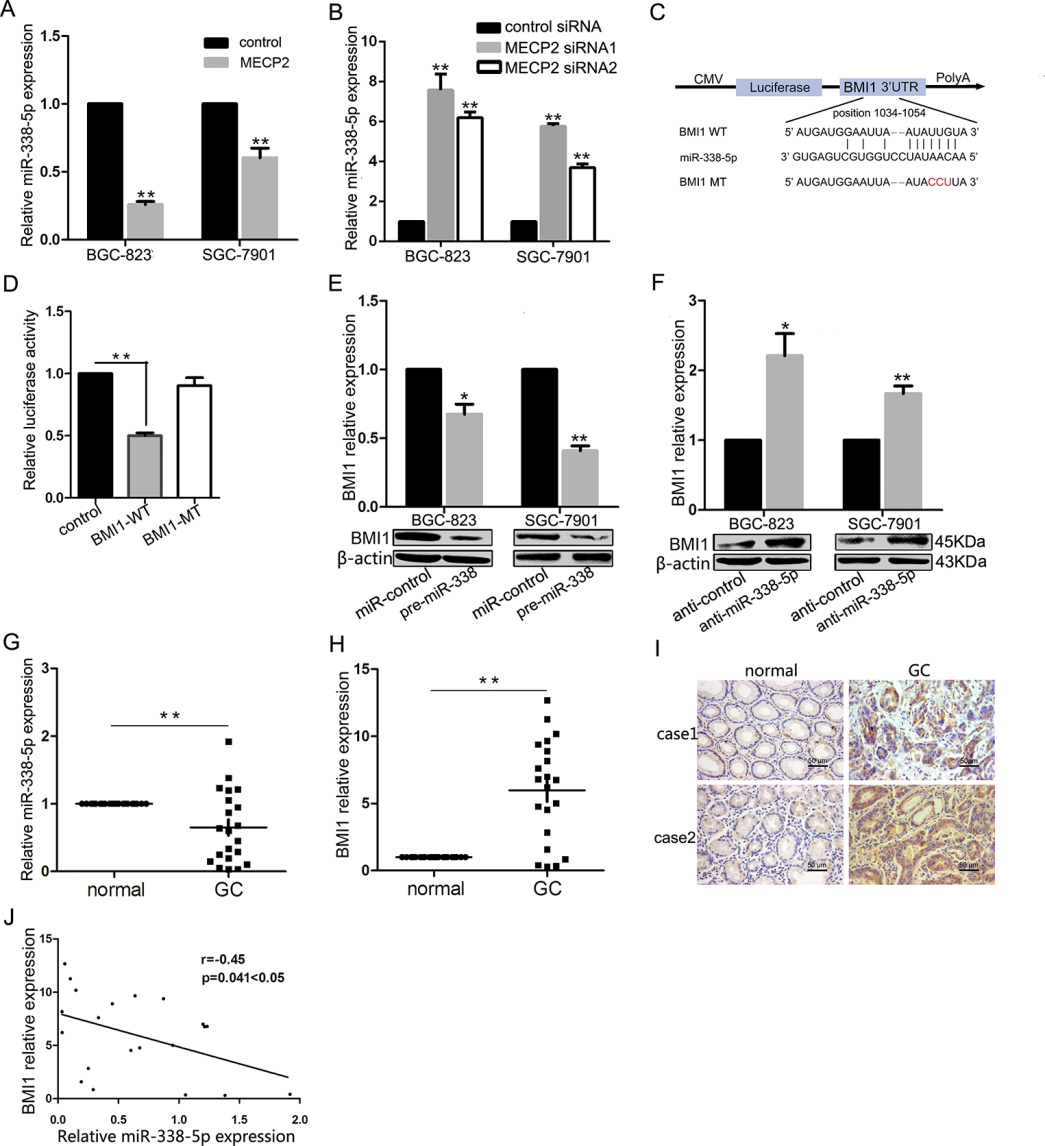
MECP2 suppressed miR-338-5p expression and miR-338-5p could target BMI1 (**A**) Ectopic expression of MECP2 suppressed miR-338-5p expression. After transfection of MECP2 vector or control vector to BGC-823 and SGC-7901 cells, expression of miR-338-5p measured by qRT-PCR. (**B**) qRT-PCR analysis of the miR-338-5p expression in BGC-823 and SGC-7901 cells transfected with MECP2 siRNA1 and siRNA2. (**C**) The target sites of miR-338-5p in 3′-UTR of BMI1 are shown as a schematic representation. The target sequence was predicted by miRNA target prediction program, TargetScan, PicTar and DIANA-microT-CDS. (**D**) Dual-luciferase reporter activity assay. Pre-miR-338 was cotransfected with target gene reporter construct (WT or MT version of pGLO constructs) or NS-control, the relative luciferase activity was normalized by calculating the ratio of firefly luciferase to the Renilla luciferase activity. All assays were performed in triplicates and repeated at least three times. (means ± SD; *P < 0.05; **P < 0.01, Student's *t* test) (**E**–**F**) mRNA and protein expression level of BMI1 were measured by qRT-PCR and Western blot analyses in BGC-823 and SGC-7901 cells transfected with pre-miR-338 vector (E) and miR-338-5p inhibitor (F). (Means ± SD; *P < 0.05; **P < 0.01, Student's *t* test). (**G**) Dysregulated miR-338-5p in GC tissues. qRT-PCR was performed to examine miR-338-5p expression in 21 paired human GC and adjacent nontumor tissues. (**H**–**I**) BMI1 mRNA and protein level were evaluated by qRT-PCR and immunohistochemistry staining for 21paired clinical specimens. (means ± SD; **P < 0.01, Student's *t* test). (**J**) Inverse correlation between miR-338-5p and BMI1 expression in GC tissues. Statistical analysis was performed using Pearson's correlation coefficient (*n* = 21, *r* = − 0.45, *P = 0.041 < 0.05).

As pattern of MECP2 depressed miR-338 was at transcriptional level, and the former result indicated that miR-338-3p was supressed by MECP2 in GC. We explored whether MECP2 could inhibit miR-338-5p, a mature microRNA originating from the opposite arms of the same pre-miRNA of miR-338-3p. Consistently, qRT-PCR showed miR-338-5p deregulation in the MECP2 overexpression vector-transfected GC cells compared to the control vector (Figure 3A), and *Mecp2* knockdown resulted in increasing miR-338-5p expression (Figure 3B). In our previous study, we used pre-miR-338 vector to show that miR-338-3p could suppress the progression of GC cells. To further extrapolate this finding and to investigate biological functional role of miR-338-5p, we first found that BMI1 was predicted to be the candidate target of miR-338-5p by using bioinformatic analysis (TargetScan, PicTar, and DIANA-microT-CDS). To determine whether miR-338-5p selectively regulated BMI1 via directly binding to the 3′-UTR region of BMI1 (Figure 3C), we cloned wild-type (WT) and mutant-type (MT) of BMI1 3′-UTR into the downstream region of pmiRGLO dual-luciferase reporter vector. As the dual-luciferase reporter assays showed that miR-338 induced 54% reduction in luciferase activity in WT vectors compared to control vectors, mutant BMI1 3′-UTR abrogated the effect of miR-338 (Figure 3D), indicating that miR-338-5p might suppress BMI1 by binding to the 3′-UTR of BMI1. In addition, to confirm the effect of ectopic expression of miR-338-5p on BMI1 levels, miR-338-5p expression was upregulated in BGC-823 and SGC-7901 GC cells on transfection with pre-miR-338 vector (Supplementary Figure 3A). It also significantly decreased BMI1 mRNA or protein levels (Figure 3E). Next, we used anti-miR-338-5p oligonucleotides to silence miR-338-5p (Supplementary Figure 3B). The results of western blot and qRT-PCR showed that on comparison with anti-miR-control, BMI1 was markedly unregulated in both GC cells transfected with anti-miR-338-5p (Figure 3F). We examined 21 pairs of GC tissues and found significant miR-338-5p deregulation in 15 of 21 (71%) samples compared to the corresponding non-tumor tissues (Figure 3G). Meanwhile, qRT-PCR assay showed that BMI1 was significantly overexpressed in GC samples compared to adjacent non-cancerous tissues. Immunohistochemistry analysis indicated that the protein level of BMI1 was highly upregulated in GC samples (Figure 3H and 3I). We also evaluated the association between miR-338-5p and BMI1 *in vivo*. Interestingly, Pearson's correlation coefficient test verified a significant inverse correlation between BMI1 mRNA and miR-338-5p expression (Figure 3J). It is notable that MECP2 mRNA expression was also inversely correlated with miR-338-5p expression, but not significantly (*P* = 0.051) (Supplementary Figure 3C). These results showed that MECP2 could also suppress miR-338-5p expression. Further, miR-338-5p is a direct inhibitor of endogenous BMI1 expression in GC cells.

### Inhibitors of miR-338-5p transfection affect cell growth and proliferation in GC cell lines

**Figure 4:**
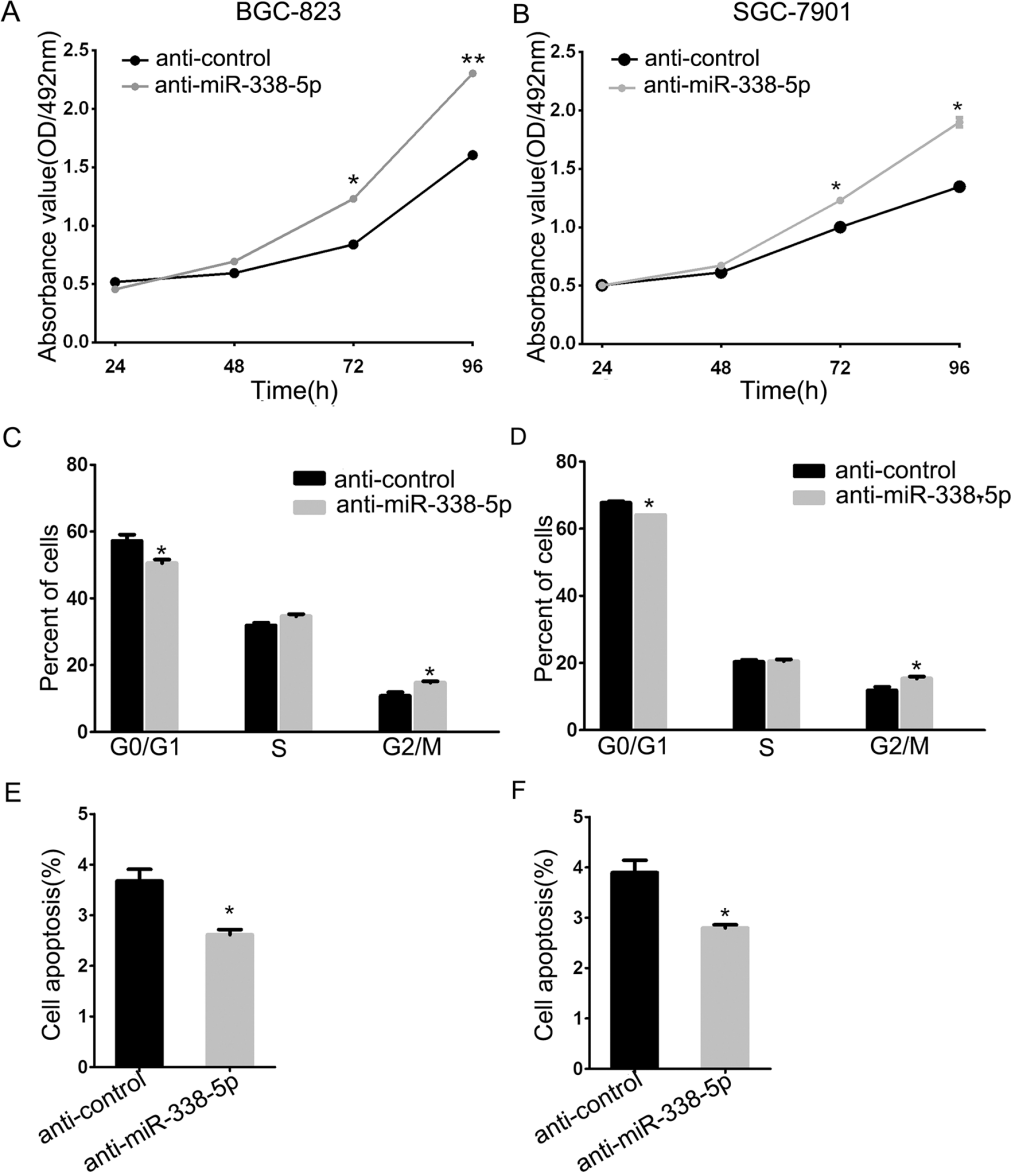
Inhibition of miR-338-5p facilitated BGC-823 and SGC-7901cells proliferation (**A**) The cell viability was determined by measuring MTT analysis at 24, 48, 72 and 96 h after transfection with miR-338-5p inhibitor and inhibitor control, respectively. (**B**) The histograms for cell cycle distribution of BGC-823 and SGC-7901 cells at 48 h after transfection of miR-338-5p inhibitor based on the flow-cytometric analysis. (**C**) Evaluation of apoptosis by annexin V-PITC (AV) and propidium iodide (PI) staining and analysis by flow-cytometry in BGC-823 and SGC-7901 cells at 48 h after transfection of miR-338-5p inhibitor, data were presented as means ± SD; *P < 0.05; **P < 0.01, Student's *t* test.

To uncover the function of miR-338-5p in the progression of GC cells, we used an inhibitor to eliminate endogenous miR-338-5p, followed by MTT assay to measure the proliferation of BGC-823 and SGC-790 cells. Ectopic silencing of miR-338-5p led to increased growth rate of these two GC cells (Figure 4A). Furthermore, cell cycle analysis was performed by flow cytometry; BGC-823 and SGC-7901 cells transfected with miR-338-5p inhibitor were found to be inclined to enter S-phase (Figure 4B). We simultaneously found that miR-338-5p can decrease the rate of early apoptosis in GC cells (Figure 4C). The early apoptosis inhibitory effect of miR-338-5p inhibitor on BGC-823 and SGC-7901 GC cells was moderate since the low endogenous apoptosis rate, which is consistent with our previous study performed using pre-miR-338, demonstrates the antitumor function of miR-338-3p. These data suggested that not only 3p but also endogenous miR-338-5p show anticarcinogenetic function on the progression of BGC-823 and SGC-7901 GC cells.

### *Bmi1* knockdown suppress cell growth and proliferation in the GC cell lines

**Figure 5:**
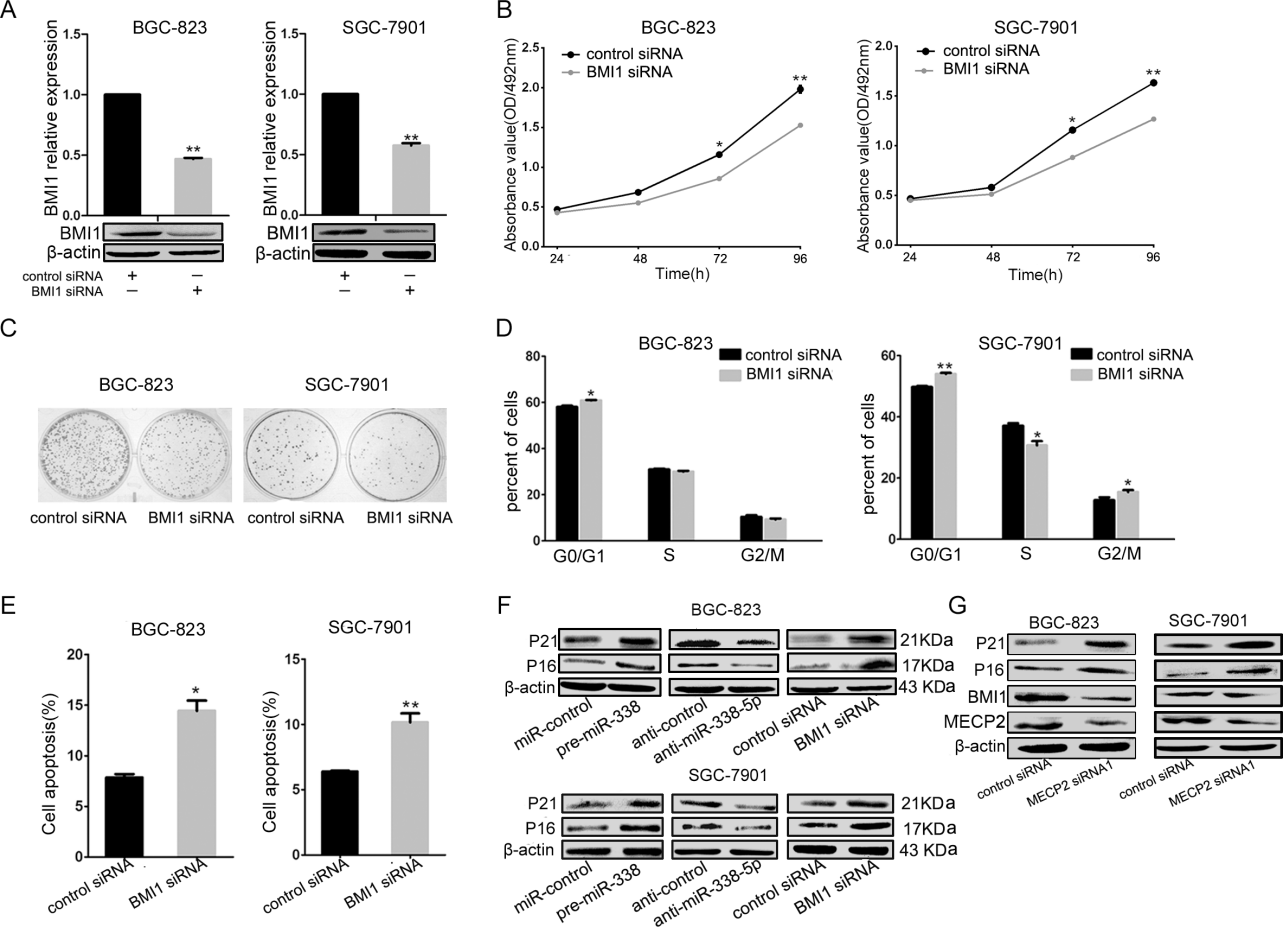
miR-338-5p inhibits cell proliferation through BMI1 (**A**) qRT-PCR and western blot analyses were performed to determine the expression level of BMI1 after transfection of BMI1 siRNA. (**B**) MTT assays was performed on 24–72 h after the transfection of BGC-823 and SGC-7901 cells with BMI1 siRNA or negtive control, data were presented as means ± SD; *P < 0.05; **P < 0.01, Student's *t* test. (**C**) Representative micrographs show the growth of BGC-823 and SGC-7901 cells was detected by colony formation assay at day 14 after transfection. (**D**) At 48 h after transfection of BMI1 siRNA to BGC-823 and SGC-7901 cells, the DNA content of PI-stained cells was analyzed by flow-cytometry. The cell number ratios are presented in bar graph (means ± SD; *P < 0.05, **P < 0.01, Student's *t* test). (**E**) Early cell apoptosis were detected by Annexin-V/propidium iodide combined labeling flow cytometry in BGC-823 and SGC-7901 cells 48 hours after transfection with BMI1 siRNA or control siRNA. Apoptotic evaluation was carried out by the percentage of apoptotic cell number in total cell number (means ± SD; *P < 0.05, **P < 0.005, Student's *t* test). (**F**) The protein expression levels of G1/S regulatory molecules in the downstream of the BMI1 were analyzed by western blot in BGC-823 and SGC-7901 cells at 48 hours after transfection with pre-miR-338 vector, miR-338-5p inhibitor, BMI1 siRNA, with control vector, inhibitor control, and control siRNA, respectively. (**G**) Expression analysis for BMI1 and downstream proteins by western blot in BGC-823 and SGC-7901 cells after transfection with MECP2 siRNA or control siRNA.

As validated by previous results, miR-338-5p directly inhibited BMI1; to investigate whether BMI1 is involved in the antitumor effect exerted by miR-338-5p, we evaluated BMI1-mediated cell proliferation, cell cycle, and cell apoptosis. First, the efficiency of RNA interference was confirmed by real-time qPCR and western blot; BMI1 can be specifically knockeddown by BMI1 siRNA in BGC-823 and SGC-7901 GC cells (Figure 5A). The MTT assay revealed that *Bmi1* knockdown caused significant inhibition of growth rate of BGC-823 and SGC-7901 cells (Figure 5B). As shown in Figure 5C, the colony formation ability of BMI1 siRNA-transfected BGC-823 and SGC-7901 GC cells was significantly lower compared to the control siRNA-transfected cells. To demonstrate whether alterations in the cellcycle mediated inhibition of cell proliferation, we analyzed the cell cycle by flow cytometry and found that *Bmi1* knockdown resulted in the accumulation of cells in the G1 phase and a decrease in the number of cells in the S phase (Figure 5D). In addition, the proportion of early apoptotic cells markedly increased in the BMI1 siRNA-transfected GC cells compared to the control siRNA-transfected GC cells (Figure 5E). Given that BMI1 is a verified target of miR-338-5p and that miR-338-5p overexpression and BMI1 silencing showed corresponding effects on BGC-823 and SGC-7901 cells, we further investigated the molecular effects and assessed relevant downstream proteins involved in the inhibition of proliferation of BMI1-mediated miR-338-5p. The protein level of P16 and P21 increased after miR-338-5p overexpression or BMI1silencing. In contrast, miR-338-5p inhibition led to a decrease in P16 and P21 levels in BGC-823 and SGC-7901 GC cells (Figure 5F). Thus, miR-338 was responsible for the inhibition of cell proliferation through the downregulation of BMI1 mediated by facilitating P16 and P21. Moreover, to better understand the mechanism underlying MECP2-induced growth activation, we performed western blot analysis for the miR-338-5p-targeting protein BMI1 and its downstream molecules, P16 and P21, after MECP2 siRNA1 transfection. BMI1 expression was decreased in BGC-823 and SGC-7901 cells (Figure 5E), while the protein expression of P16 or P27 was significantly upregulated. Thus, we identified an MECP2/miR-338-5p/BMI1 axis function in GC.

### *Mecp2* knockdown inhibits cell growth *in vivo*

**Figure 6:**
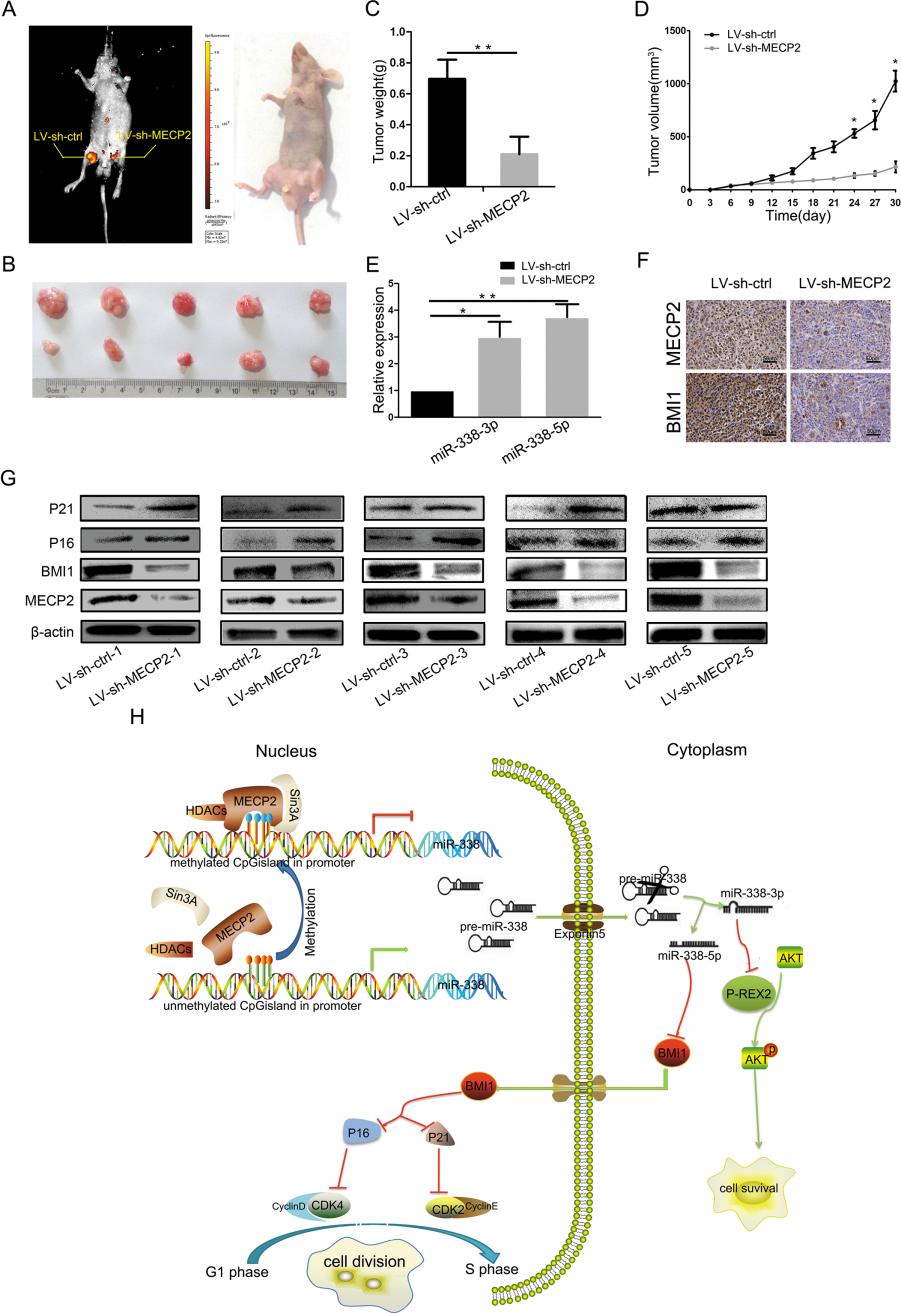
silencing MECP2 inhibited GC growth *in vivo.* (**A**) At day 30, tumor growth was measured by *in vivo* bioluminescence imaging (uper portion). The flanks were injected with SGC-7901cells infected with LV-sh-ctrl (leftflank) and LV-sh-MECP2 infected SGC-7901 cells (right flank) in 5 nude mice, respectively. (**B**) The gross morphology of tumors (**C**) The mice were anesthetize and sacrificed at the experimental endpoint and tumors infected with LV-sh-MECP2 and LV-sh-ctrl were weighted at day 30 after the initial injection. Data were represented as mean ± SD, **P < 0.01. (**D**) Tumor growth curves of tumor volume was formed every 3 days for 27 days (*n* = 5). (**E**) The expression levels of miR-338-3p and miR-338-5p were detected by qRT-PCR analysis in the tumor tissues from the animals. (**F**) IHC staining of MECP2 and BMI1 in the tumor xenografts(means ± SD; *P < 0.05, **P < 0.01, Student's *t* test). (**G**) Protein level of MECP2, BMI1 and G1/S regulatory molecules in BMI1 downstream detected by western blot. (**H**) Proposed model for MECP2 promotes the cell proliferation and gene regulatory effect of miR-338 mediates by MECP2 on the BMI1 or P-REX2 in GC. MECP2 occupation of miR-338 promoter CpG island undergone methylated modification and depresses the miR-338-3p and 5p expression. MiR-338-3p could mediate the antiproliferative via targeting P-REX2 thereby affect AKT pathways; miR-338-5p regulates cell cycle through targeting BMI1 and effect cell cycle related molecules in GC.

To further demonstrate the impact of MECP2 on the growth of GC cells *in vivo*, we used lentiviral vectors to stably knockdown Mecp2 in BGC-823 cells. LV-sh-MECP2- and LV-sh-ctrl-infected SGC-7901 cells were subcutaneously injected into either posterior flank of the same nude mouse. Xenografts were measured every 3 days for 4 weeks. Tumor growth was significantly suppressed by LV-sh-MECP2, compared to LV-sh-ctrl (Figure 6A and 6B). On day 30, the average volume and weight of LV-sh-MECP2-treated tumors were much smaller than those of control tumors (Figure 6C and 6D). Furthermore, we evaluated miR-338-3p and miR-338-5p expression in the tumors excised from animals by qRT-PCR, the expression of the miR-338-3p and miR-338-5p was both increased (Figure 6E). We assessed MECP2 and BMI1 protein levels by Immunohistochemistry and western blot, and obtained results consistent with those of the *in vitro* assay, that is, decreasing MECP2 caused BMI1 reduction. Western blot also showed that both P16 and P21 were upregulated in LV-sh-MECP2-treated tumors compared to LV-sh-ctrl-treated tumors (Figure 6F and 6G). These data together with previous results indicated that MECP2 can promote GC cell proliferation through suppressing miR-338 and activate PREX-2 and BMI1 expression (Figure 6H).

## DISCUSSION

In past few years, many epigenetic targets have been identified that are involved in different types of cancers such as GC, ovarian cancer, colorectal cancer, breast cancer, lung cancer, and colon cancer [5, 11, 30–32]. In GC, genetic and epigenetic alterations are involved in the pathogenesis of cancerous lesions. Tumor suppressors such as *CDKN2A*,*MLH1*, *CDH1*, *LOX*, and APC have been shown to undergo methylation in GC [33]. Human proteins, namely, MECP2 (this protein), MBD1, MBD2, MBD3, and MBD4 comprise a family of nuclear proteins related to the presence of a methyl-CpG-binding domain (MBD). Except MBD3, all MBDs are capable of specifically binding to methylated DNA. As MECP2 binds to methylated DNA, it recruits the co-repressors Sin3A and histone deacetylases (HDACs) 1 and 2 via its central transcription repressiondomain and repressesgenetic transcription [34]. MECP2 is originally and widely studied in neuronal systems. Further studies revealed that MECP2 participates in a wide range of cellular processes such as fibrosis [35] and tumorigenesis [18]. MECP2 may also act to translate various environmental experiences into lasting epigenetic changes in some cancer repressor genes. This hypothesis has been confirmed by our finding. First, we analyzed MECP2 expression at the protein and mRNA levels. MECP2 is significantly overexpressed in GC samples compared to normal tissues (Figure 1). Furthermore, MECP2 silencing results in inhibition of cell proliferation, cell cycle arrest during G1/S transition, and increased early apoptosis in GC cells *in vitro* (Figure 1G and 1H, Supplementary Figure 1). Also, ectopic expression of MECP2 in SGC-7901 cells inhibited the proliferation of gastric tumors *in vivo* (Figure 6). MECP2 has been verified to be highly upregulated in GC [36], which is in accordance with the findings for prostate cancer and hepatocellular carcinoma [17, 21]. However, there is lack of knowledge about the mechanisms underlying MECP2 functions in tumorigenesis. In 2003, Keri Martinowich *et al.* reported that MECP2 binds to the methylated CpG sites closed to promoter III of *Bdnf* in resting neurons [37]. The presence of MECP2 within the promoter region of *Gtl2/Dlk1*-imprinted domain was first identified in a study on RTT [38]. In addition, MECP2 could inhibit the tumor growth suppressor *C/EBPδ* by binding to the *CpG*-dense C/EBPδ promoter [39]. Carvell T *et al* showed the presence of three CpG islands in the *P14^ARF^/P16I^NK4A^* locus in a series of normal cancer cell lines. MECP2 could bind to the methylated CpG islands in both promoters and exons. However, binding only to the promoters could interfere with transcription [40]. Thus, MECP2 plays a critical role in cancer progression, and we aim to present a novel mechanism underlying these positive effects of MECP2 on tumorigenesis in GC.

Abundant miRNAs regulate a wide range of biological processes in cancer research. The expression of some miRNAs was involved in epigenetic mechanism such as miR-129 and miR-196b [23, 24, 41]. Furthermore, *Aatk*, the hostgene of miR-338, was confirmed to be a tumor suppressor gene, whose expression was regulated by methylation of this CpG island located in its promoter [26]. Consistent with this study, we treated the GC cells with 5-Aza, a DNA methyltransferase inhibitor, and found that the expression of miR-338-3p and pre-miR-338 was significantly upregulated (Figure 2A). Studies have found that the CpG island promoters of some miRNAs were inactive when MECP2 occupied their CpG islands [15, 42]. We found that the expression of both miR-338-3p, pre-miR-338 and miR-338-5p was regulated by MECP2 (Figure 2, Figure 3A and 3B), and that the method of MECP2 binding on the verified CpG island of miR-338 promoter was identified by chromatin immunoprecipitation(CHIP)(Figure 2G). These results can help us understand that the mechanism underlying MECP2-induced regulation of miR-338 is transcriptional and epigenetic-related. Importantly, recent studies have showed that MECP2 posttranscriptionally regulates miRNA expression. MiRNA-134 is subjected to this processingmachinery [43]. However, miR-338 was not specified in the study.

As shown by our previous study, miR-338-3p can suppress GC progression through a PTEN-AKT axis by targeting *P-rex2* [28]. Corresponding to the effect of *Mecp2* knockdown, miR-338-3p caused inhibition of proliferation, blockade of G1/S transition, and acceleration of apoptosis in GC cells. To verify the positive effects of MECP2 on gastric tumorigenesis via miR-338 suppression, we tested the identified target of miR-338-3p, P-REX2, and the PTEN-AKT axis-correlated proteins by western blot. It was found that silencing MECP2 deregulated P-REX2 and phosphorylated AKT at serine 473 (Figure 2H). The rescue experiment showed that miR-338-3p inhibition counterbalanced the effect of *Mecp2* knockdown in GC cells during cell proliferation and apoptosis (Figure 2I and 2J). Our results highlight that the relationship between MECP2 and miR-338 interaction plays an important role in GC progression. Furthermore, Na Huang *et al*. have independently identified that miR-338-3p is deregulated and that it act as a tumor suppressor in GC by targeting ZEB2 and MACC1/Met/Akt signaling [44]. MiR-338-3p serves as a tumor suppressor in GC, and both miR-338-5p and miR-338-3p originate from pre-miR-338; however, the reports about the function of miR-338-5p in GC are few. Studies by two independent groups(Qi Xue et al and Chunxi Liu et al) and our former studies have taken advantage of pre-miR-338 in study of anti-tumorigenetic property of miR-338-3p [28, 45, 46]. MiR-338-5p can be spliced from pre-miR-338 and miR-338-5p was upregulated similar to that observed in pre-miR-338-transfected cancer cells; therefore, it is reasonable that miR-338-5p participates in cancer progression. In previous studies, to the best of our knowledge, the function of miR-338-5p in tumorigenesis was reported in only one study that regarded miR-338-5p as an oncomir inducing cell migration by suppressing PIK3C3 expression and autophagy in colorectal cancer [47]. Contradictory to previous observations, we further confirmed that miR-338-5p is downregulated in the GC tissues and believed that miR-338-5p functions as a tumor suppressor. Corresponding to former gain-of-function studies, the inhibition of miR-338-5p can also affect the proliferation, cell cycle, and apoptosis of GC cells *in vitro*. Because of the different types of cancer, miR-338-5p offers different functions to discuss. In addition, our luciferase assay showed that BMI1 is a direct target of miR-338-5p. Overexpression of miR-338-5p significantly suppressed BMI1 protein expression and the BMI1-medimated protein such as P16 and P21 were induced in GC cells. Our results demonstrated that miR-338-5p could be suppressed in cell proliferation by targeting BMI1 in GC.

BMI1, (B lymphoma Mo-MLV insertion region 1 homolog) has been reported to function as an oncogene by regulating *p16^Ink4a^* and *p14^Arf^*, which are cell cycle inhibitor genes. A recent study showed that BMI1 plays an important role in limiting genomic instability by positive regulation of *p21* [48, 49]. BMI1 is frequently overexpressed in human myeloma, prostate cancer, and lung cancer [48–50]. It has been identified that some miRNAs can directly target BMI1, including miR-203, miR-218, and miR-429 [50, 51]. However, there are limited reports about the expression and functions of BMI1 in the proliferation of GC. We verified BMI1 was upregulated in GC tissues by RT-PCR and immunohistochemical staining, and that miR-338-5p was inversely correlated to BMI1. Importantly, silencing BMI1 showed the same cellular and molecular effect on GC cells as did miR-338-5p overexpression and *Mecp2* knockdown. Furthermore, our former study indicated that MECP2 could exert indirect regulation on P-REX2 by miR-338-3p, accordingly, the current study showed the inhibition effect of MECP2 on miR-338-5p that target to BMI1 could also lead to the upregulation of P16 and P21.

The indirect regulation of MECP2 exerted by miR-338-3p on the target gene P-REX2, decreased BMI1 expression and led to P16 and P21 upregulation *in vitro*. Increased miR-338-3p and 5p, BMI1 inhibition, P16 and P21 upregulation were induced by Mecp2 knockdown *in vivo*.

Above all, we hereby provide a new mechanism that is utilized by MECP2 to promote GC proliferation by the cell signaling pathways of MECP2/miR-338/P-REX2 or BMI1, which may be a potential therapeutic strategy for the treatment of GC in future.

## MATERIALS AND METHODS

### GC tissues

Paired GC and adjacent non-tumor gastric tissues were obtained from 21 patients who had undergone surgical gastric resection without preoperative treatment at the First Affiliated Hospital of Xi'an Jiaotong University. Tissue samples were immediately snap frozen in liquid nitrogen until RNA extraction. Both tumor and non-tumor tissues were histologically confirmed. Informed consent was obtained from each patient and was approved by the Institute Research Ethics Committee at Cancer Center, xi'an Jiao tong University. The basic information of all patients were shown in Supplementary Table 1.

### Cell lines and transfer

Human gastric cancer BGC-823, SGC-7901 GC cell lines were obtained from the Cell Bank (Shanghai Genechem Co., Ltd., Shanghai, China). Cell lines BGC-823, SGC-7901 were maintained in 1640 medium (1640; PAA Laboratories GmbH) supplemented with 10% FBS (PAA Laboratories GmbH) and cultured in a humidified 5% CO2 incubator at 37°C. Cell lines were transfected with Lipofectamine 2000 (Invitrogen, Carlsbad, CA, USA) following the manufacturer's protocol.

### Plasmid construction and oligonucleotides

Human miR-338 precursor was in frame subcloned into pcDNA6.2GW/eGFP which was purchased from Invitrogen. Synthetic primary transcript of miR-338 (AuGCT DNA SYN Biotechnology Co.Ltd, Beijing, china) designed into EcoRI and HindIII enzyme restriction sites to facilitate cloning into the vector. The complimentary sites in 3′UTR of BMI1 for miR-338 were synthesized (AuGCT DNA-SYN Biotechnology Co. Ltd Beijing, china) and cloned into pmirGLO vector at Sac1 and Xho1 sites (Promega, Madison, USA) and named BMI1-WT. While the partial mutant BMI1 3′UTR sequences were also inserted into pmirGLO vector and named BMI1-MT. GV141 vector containing cDNA of MECP2 was purchased from GeneChem. The inhibitor of miR-338-3p and miR-338-5p, small interfering RNA (siRNA) targeting MECP2 and BMI1 were purchased from GenePharma. All sequences are shown in Supplementary Table 2.

### RNA extraction, cDNA synthesis and qRT-PCR

Total RNA was extracted from cell lines or frozen tissues using TRIzol reagent (Invitrogen, Carlsbad, CA, USA). For mRNA analyses, the first-strand cDNA was synthesized according to the manufacturer's protocol (Toyobo, Osaka, Japan). while for quantification of miR-338-3p, miR-338-5p and pre-miR338, miRNA was reverse transcribed using miRNA-specific primers. The cDNA was analyzed by a LightCycler^®^ 480 (Roche Diagnostics, Tokyo, Japan) and subjected to 45 cycles of amplification using a standard SYBR Green PCR Master Mix (Toyobo, Osaka, Japan). Relative expression levels were normalized to control. The endogenous U6 snRNA or β-actin was chosen as the internal control for miRNA and mRNA level, respectively, calculations used the average Ct from triplicate assays. The relative expression of genes (pre-miR338, miR-338-3p, miR-338-5p, U6, MECP2, BMI1, β-actin) was calculated with the 2^−(ΔΔCt)^ method. The primers used are listed in Supplementary Table 3.

### MTT assay

Gastric cancer BGC-823 and SGC-7901 cells were seeded into 96-well plates at 3000 cells/well in 200 μl/well culture medium. At 24, 48, 72 and 96 h after transfection, cells were washed with warm 1640 and MTT (3-(4,5-dimethyl-2-thiazolyl)-2,5-diphenyl-2-H-tetrazolium bromide) (Sigma) working solution was added into wells. Cells were incubated at 37°C for 4 hour and then supernatant was discarded and replaced with 150 μl Dimethyl sulfoxide(DMSO) to solubilized the converted dye. Cell viability was measured on FLUO star OPTIMA (BMG). Each assay was repeated in triplicate.

### Colony formation assay

At 24 h post-transfection, cells were harvested and reseeded in 6-well plates at 1000 cells/well. After grown for 2 weeks, Colonies were stained with 0.1% crystal violet, counted and normalized to the control group.

### Cell cycle assay

The BGC-823 and SGC-7901 cells were seeded into 6-well plates at 1 × 10^6^. At 48 h after transfection, cells were harvested by trypsinization, washed with phosphate-buffered saline (PBS) and then fixed with 70% ethanol at 4°C overnight. Cells were centrifuged at 1500 rpm for 5min, washed twice again and added 150 μl 0.1 mg/ml Rnase A and 0.05 mg/ml propidium iodide (PI) each to incubate at 4°C for 30 minutes, then cells populations in G0–G1, S, and G2-M phase were analyzed by flow cytometer (FACSort; Becton).

### Cell apoptosis assay

The cells were seeded into 6-well plates at 1 × 10^6^ cells per well. After transfection for 48 h, cells were harvested and washed. The apoptosis populations were examined by flow cytometer (FACSort; Becton) using Annexin-VFITC ApoptosisDetection Kit (Invitrogen).

### Dual luciferase reporter assay

BGC-823 cells were seeded in 96-well plates(3000 cells per well) one day

before transfection. After plating, the pre-miR-338 vector cotransfected with wild or mutated 3′-UTR BMI1 reporter constructs and a blank pmirGLO Dual-Luciferase as a positive control into cells. Luciferase activity was detected 24 h later using the Dual-Glo luciferase assay system (Promega, Fitchburg, WI, USA) according to the manufacture's protocol.

### Western bolt analysis

Protein samples of tissue and transfected cells were harvested with RIPA buffer (Sigma-Aldrich). Concentration of protein lysate was quantified by using bicinchoninic acid protein assay, BCA(Sigma). Equal amounts of Proteins were resolved with 7–10% SDS-PAGE gel, subsequently transferred to the methanol-activated PVDF membrane (Millipore, Beijing, China). The membranes were blocked with 5% non-fat milk in Tris-buffered saline Tween-20 (TBST) for 2 hr at room temperature (RT) and then incubated overnight with the following antibodies: anti-MECP2, anti-P-REX2, anti-P473-AKT (Abcam, diluted 1/1000), anti-BMI1, anti-P16, anti-p21 (Cell Signaling Technology, diluted 1/1000) and anti-β-actin (Santa Cruz 1/1000) which were chosen as a loading control, followed by secondary HRP-conjugated anti-rabbit or anti-mouse (Santa Cruz).

### Immunohistochemistry(IHC)

The human tumor tissues and Xenografts excised from animals were made into FFPE tissue samples. The samples were sectioned at 5 μm thickness, and the sections were pretreated with deparaffinized, hydrated, microwave, blocked, and incubated using primary antibodies (MECP2 and BMI1) at 4°C overnight. The sections were then incubated with secondary antibody. Detection was performed by 3, 3′-diaminobenzidine(DAB)(Sigma) and hematoxylin. Staining intensity was assessed by Leica Q550 image analysis system.

### Chromatin immunoprecipitation assay

Chromatin immunoprecipitation assay(CHIP) was performed according to the methods described previously [20]. Briefly, BGC-823 cells were pretreated with cross-linked, ultrasonication. Sheared chromatin was immunoprecipitated.

with anti-MECP2 or IgG antibody (Abcam) overnight at 4°C. The DNA was purified from the immunocomplexes and used as template for ChIP-PCR assay. the primers for quantitative real-time PCR are listed in Supplementary Table 3.

### Tumor xenograft model

4–6week-old male BALB/C nude mice were obtained from Animal Center of Xi'an Jiao Tong University and housed in pathogen-free room. SGC-7901 cells were infected with LV-sh-MECP2 and LV-sh-ctrl. 1 × 10^7^ stable gastric cells (LV-sh-MECP2 and LV-sh-ctrl) were resuspended with100 μml PBS were injected subcutaneously into the both posterior flank of nude mice. Tumor size was measured every 3 days for 4 weeks and monitored by bioluminescent imaging (Xenogen IVIS Spectrum USA). Tumor volumes were calculated by Tumor volume was calculated as length × width^2^ × ½ mm^3^.

### Statistical analysis

Data were presented as mean ± SD of three independent experiments at least, the Student's *t* test was used for comparisons of 2 independent groups. All statistical analysis was performed using SPSS13.0 software (SPSS Inc.). Spearman's correlation was used to explore the association between miR-338-5p and BMI1 expression. All tests were two-sides and differences were considered statistically significant at *P* < 0.05.

## SUPPLEMENTARY MATERIALS FIGURES AND TABLES


