# Supplementary material for: MECP2 promotes the growth of gastric cancer cells by suppressing miR-338-mediated antiproliferative effect
Source: Oncotarget. 2016 May 6;7(23):34845–59. doi: 10.18632/oncotarget.9197 (PMC5085194; doi:10.18632/oncotarget.9197)
Supplement: Supplementary file 1 [file oncotarget-07-34845-s001.pdf]

MECP2 promotes the growth of gastric cancer cells by suppressing miR-338-mediated antiproliferative effect

Supplementary Materials

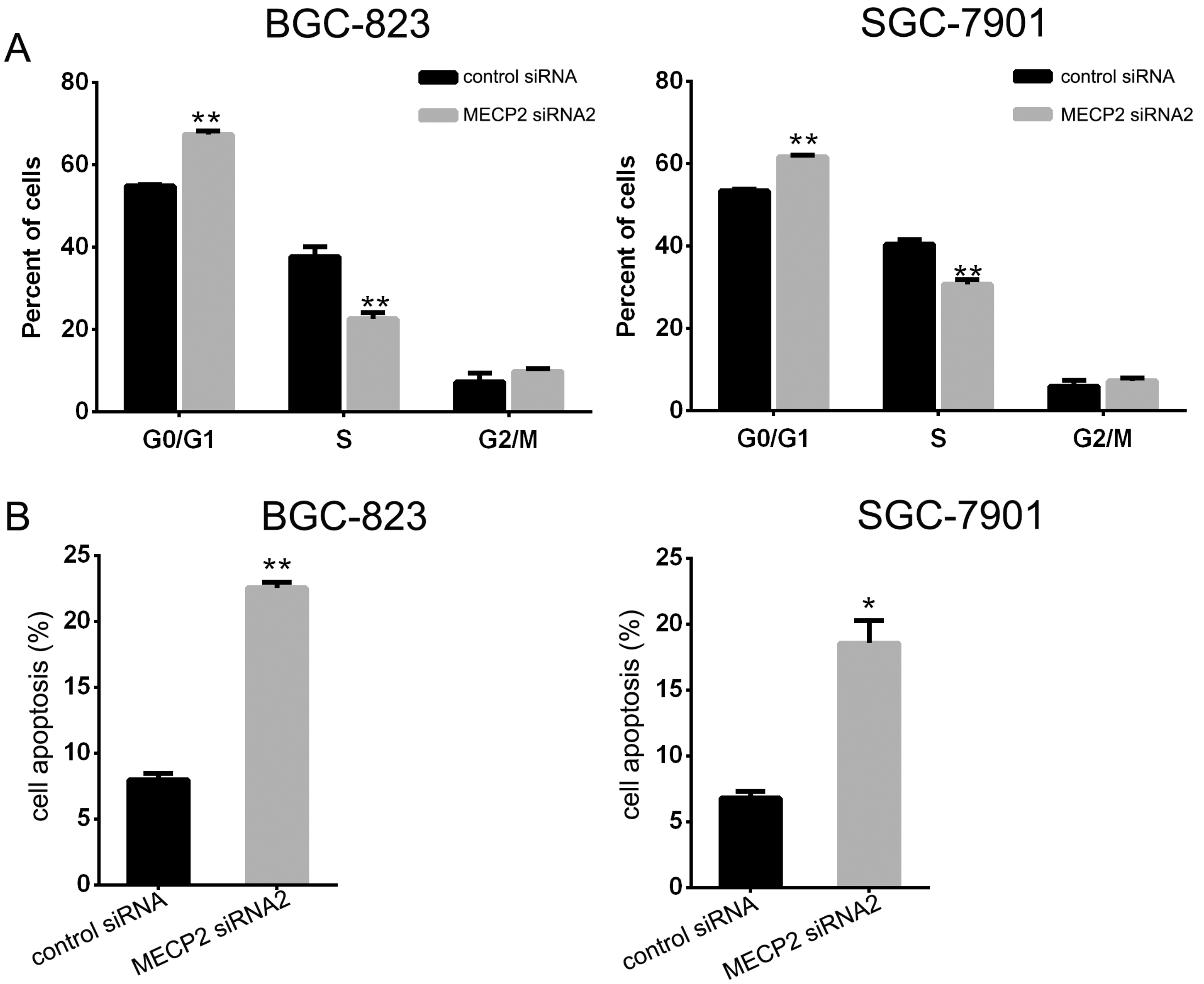

Supplementary Figure S1: (A) Cell cycle distribution of BGC-823 and SGC-7901 cells treated with MECP2 siRNA2 after 48 h based on the flow-cytometric analysis. (**B**) Apoptosis rate of BGC-823 and SGC-7901 cells was examined by Annexin V staining and flow cytometry at 48 h after transfecting with MECP2 siRNA2, representative results of 3 independent experiments were shown. Data shown are mean ± SD value. (*$P < 0.05$, **$P < 0.01$, Student's *t*-test).

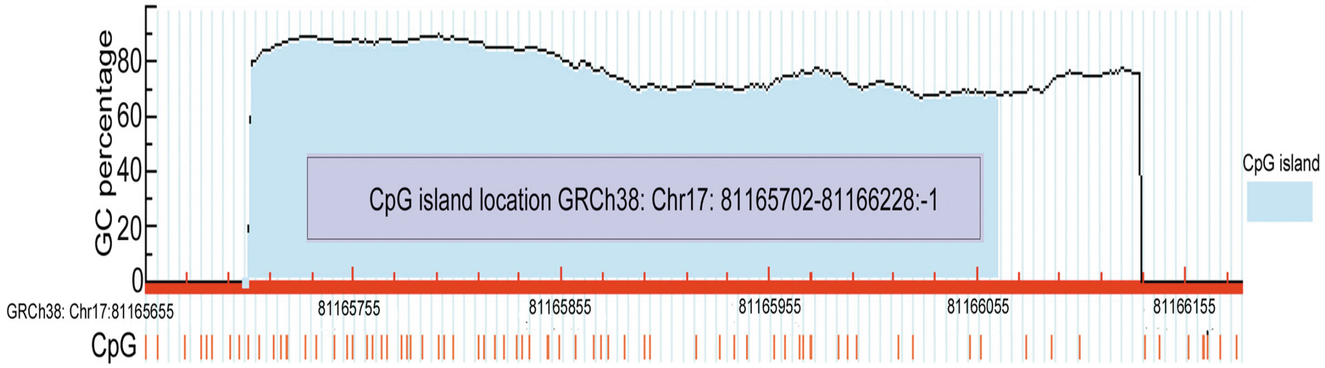

Supplementary Figure S2: The CpG island of miR-338 promoter was predicted by methprimer according to the manufacturer's protocol. Graphic view showing sequence features including GC percentage (Y-axis), CpG island (blue region), CpG site (red bar).

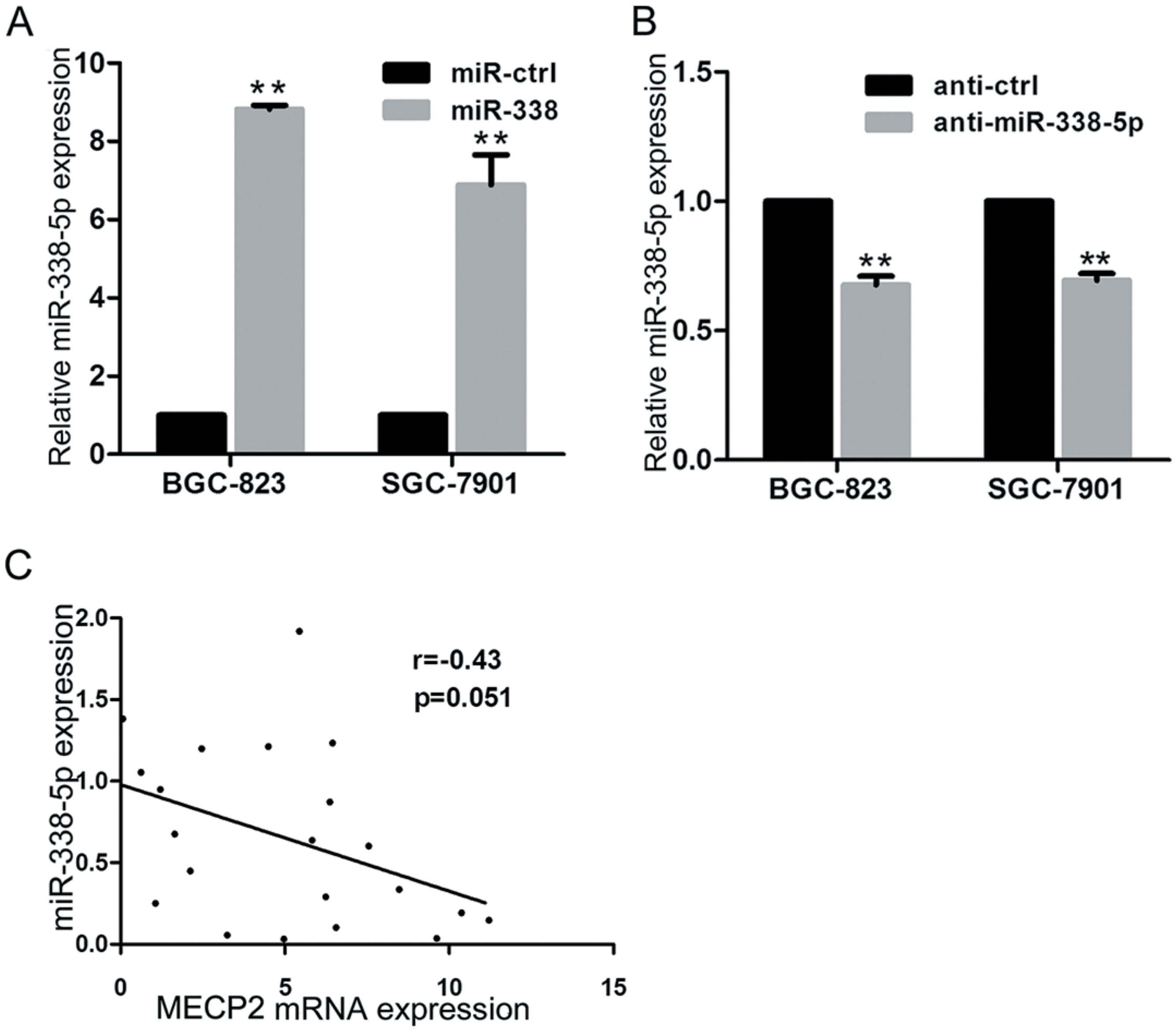

Supplementary Figure S3: (A–B) qRT–PCR were performed to determine the expression level of miR-338-5p after transfection of pre-miR-338 or miR-338-5p inhibitor. (**C**) Pearson's correlation coefficient verified the correlation between MECP2 mRNA and miR-338-5p expression ($r = 0.43$, $P = 0.051$).

Supplementary Table S1: Patient characteristics and clinicopathologic

Patient No.	Sex	Age	pTNM stage
1	Female	55	T4N2M0
2	Male	61	T3N0M0
3	Male	61	T2N0M0
4	Male	66	T2N0M0
5	Male	48	T2N0M0
6	Male	73	T2N0M0
7	Male	68	T2N0M0
8	Male	71	T3N1M0
9	Male	46	T3N0M0
10	Male	65	T3N2M0
11	Male	61	T3N1M0
12	Male	27	T3N0M0
13	Male	74	T2N0M0
14	Male	67	T3N0M0
15	Male	63	T4N2M0
16	Male	74	T3N0M0
17	Male	61	T3N0M0
18	Male	54	T3N2M0
19	Male	55	T2N0M0
20	Female	59	T2N0M0
21	Male	61	T3N0M0

Supplementary Table S2: Oligonucleotides and interfering RNA target sequences

Gene name	Sense strand	Antisense strand
**Pre-miR-338**	AATTCTCTCCAACAATATCCTGGTGC TGAGTGATGACTCAGGCGACTCCAG CATCAGTGATTTTGTTGAAGAA	AGCTTTCTTCAACAAAATCACTGA TGCTGGAGTCGCCTGAGTCATCACT CAGCACCAGGATATTGTTGGAGAG
**miR-338-3p inhibitor**	CAACAAAATCACTGATGCTGGA	
**miR-338-5p inhibitor**	CACTCAGCACCAGGATATTGTT	
**Inhibitor control**	CAGTACTTTTGTGTAGTACAA	
**BMI1-wt**	ATGATGGAATTAATATTGTA	TACAATATTAATTCCATCAT
**BMI1-mut**	ATGATGGAATTAATACCTTA	TAAGGTATTAATTCCATCAT
**MECP2 siRNA1**	GCUUAAGCAAAGGAAAUCUTT	AGAUUUCCUUUGCUUAAGCTT
**MECP2 siRNA2**	GCUUCCCGAUUAACUGAAATT	UUUCAGUUAAUCGGGAAGCTT
**BMI1 siRNA**	GCAGAAAGCACGAACAATT	UUGUUCGAUGCAUUUCUGCTT
**Control siRNA**	UUCUUCGAACGUGUCACGUTT	ACGUGACACGUUCGGAGAATT
**sh-MECP2**	TGCTTAAGCAAAGGAAATCTCTC GAGAGATTTCCTTTGCTTAAGCTTTTTTC	GAAAAAAGCTTAAGCAAAGGAAA TCTCTCGAGAGATTTCCTTTGCTTAAGCA
**sh-ctrl**	TTTCTCCGAACGTGTCACGTCTCG AGACGTGACACGTTCGGAGAA TTTTTTC	GAAAAAATTCTCCGAACGTGTCAC GTCTCGAGACGTGACACGTTCGGAGAAA

Supplementary Table S3: Primer for qRT-PCR and Sequences of recombinant plasmids

Gene name	Forward	Reverse
**MECP2**	GCCGAGAGCTATGGACAGCA	CCAACCTCAGACAGGTTTCCAG
**miR-338-3p**	ATCCAGTGCGTGTCGTG	TGCTTCCAGCATCAGTGAT
**miR-338-5p**	ATCCAGTGCGTGTCGTG	TGCTAACAATATCCTGGTG
**BMI1**	CCATTGAATTCTTTGACCAGAA	CTGCTGGGCATCGTAAGTATC
**U6**	GCTTCGGCAGCACATATACTAAAAT	CGCTTCACGAATTTGCGTGTCAT
**β-actin**	CCAACCGCGAGAAGATGA	CCAGAGGCGTACAGGGATAG
**pre-miR-338**	TCTTCAACAAAATCACTGAT	TCTCCAACAATATCCTGGTG
**MECP2 binding site primer**<br>**Forward:** GCCATGTCGTCGTCCTTCTTCA<br>**Reverse:** CCAGGGGCATCACGTCCG		
**miR-338-3p specific stem-looped RT primer:**<br>GTCGTATCCAGTGCGTGTCGTGGAGTCGGCAATTGCACTGGATACGACCAACAAA		
**miR-338-5p specific stem-looped RT primer:**<br>GTCGTATCCAGTGCGTGTCGTGGAGTCGGCAATTGCACTGGATACGACCACTCAG		
**Pre-miR-338 specific RT primer:**<br>TCTTCAACAAAATCACTGATGCT		
